# Desquamative Gingivitis: A Narrative Review of Practical Diagnostic Approach for General Dental Practitioners

**DOI:** 10.7759/cureus.114545

**Published:** 2026-08-14

**Authors:** K Anitha, Viswanathan Shanthi, M Ebenezer, G Harshini, Thangadurai Maheswaran, Vadivel Ilayaraja

**Affiliations:** 1 Periodontics, Government Dental College and Hospital, Cuddalore District, Chidambaram, IND; 2 Oral Pathology and Microbiology, Tamil Nadu Government Dental College and Hospital, Chennai, IND; 3 Periodontics, Adhiparasakthi Dental College and Hospital, Melmaruvathur, IND; 4 Oral and Maxillofacial Pathology, Sri Ramakrishna Dental College and Hospital, Coimbatore, IND; 5 Oral and Maxillofacial Pathology, Adhiparasakthi Dental College and Hospital, Melmaruvathur, IND; 6 Oral and Maxillofacial Pathology, Vivekanandha Dental College for Women, Namakkal, IND

**Keywords:** desquamative gingivitis, differential diagnosis, direct immunofluorescence, erythematous gingiva, general dental practice, mucous membrane pemphigoid, oral lichen planus, pemphigus vulgaris

## Abstract

Desquamative gingivitis (DG) is a clinical sign characterized by gingival erythema, epithelial desquamation, and erosion rather than a discrete diagnosis. It most commonly reflects underlying oral lichen planus, mucous membrane pemphigoid, or pemphigus vulgaris. As affected patients typically present to general dental practitioners, timely recognition and structured triage are essential for effective management. However, diagnostic delays remain common and can extend the management period by several months. This narrative review synthesizes the current evidence on the clinical presentation, epidemiology, etiology, differential diagnosis, diagnostic workup, and management pathways relevant to general dentistry for this condition. Key findings indicate that a staged clinical history and examination can substantially narrow the differential diagnosis prior to biopsy, that histopathology combined with direct immunofluorescence offers superior diagnostic yield compared with histopathology alone, and that biopsy technique and site selection, favoring perilesional or uninvolved tissue and atraumatic sampling methods, meaningfully affect the diagnostic accuracy. Chronic ulcerative stomatitis and lichen planus pemphigoides are important but frequently overlooked mimics of more common causes. While disease-specific pharmacotherapy generally requires specialist oversight, professional oral hygiene reinforcement and gentle periodontal care delivered by general dental practitioners produce consistent, evidence-supported improvements in pain and lesion activity across all underlying diagnoses. A structured, practical approach to recognition, biopsy planning, and prompt referral can shorten the diagnostic pathway and improve long-term outcomes in patients with this challenging clinical condition.

## Introduction and background

Desquamative gingivitis (DG) was first proposed in 1932 to describe a clinical triad of erythema, epithelial desquamation, and erosion or blistering of the marginal and attached gingiva. It remains a frequently encountered but under-recognized presentation in general dental practice [1,2]. Rather than constituting a diagnosis in itself, DG is a shared clinical phenotype produced by a range of underlying mucocutaneous and systemic diseases, most commonly oral lichen planus, mucous membrane pemphigoid, and pemphigus vulgaris, which together account for the substantial majority of reported cases [1,3]. Because patients with DG typically present first to a general dental practitioner (GDP) rather than a dermatologist or oral medicine specialist, the GDP occupies a critical, often first-contact position in a diagnostic pathway that has historically been prolonged and inefficient, with studies documenting professional diagnostic delays averaging over seven months and involving consultations with multiple clinicians before a definitive cause is identified [4,5].

The clinical significance of accurate and timely recognition extends beyond oral discomfort. Several conditions underlying DG pose a significant systemic risk. Mucous membrane pemphigoid can produce scarring at ocular and other extraoral mucosal sites with a risk of blindness, while pemphigus vulgaris can behave as a severe systemic mucocutaneous disease requiring urgent systemic immunosuppression [6,7]. Moreover, the chronic pain characteristic of DG frequently discourages effective oral hygiene, creating a secondary risk pathway for periodontal breakdown and tooth loss, which compounds the primary disease [8]. Despite this clinical importance, a persistent knowledge gap among GDPs regarding the differentiation, appropriate biopsy technique, and referral thresholds for DG remains, prompting calls for clearer diagnostic algorithms and structured clinical guides specifically tailored to non-specialist practice [1,3]. This narrative review aims to synthesize the current evidence on the clinical features, epidemiology, etiology, differential diagnosis, diagnostic workup, management, and referral pathway for DG, with a practical focus on equipping GDPs to recognize this presentation efficiently and initiate an appropriate diagnostic and therapeutic course.

## Review

Clinical features and epidemiology

DG is a clinical descriptor, not a diagnosis, applied to gingiva showing erythema, epithelial desquamation, erosion, and occasional vesiculobullous changes affecting the marginal and attached tissues [1,5]. The condition typically spares nothing consistently; lesions may be localized to a few papillae or extend across the full width of the attached gingiva, most classically on the labial aspect of the anterior maxillary and mandibular teeth [3,5]. Gentle lateral pressure on apparently normal gingiva can separate the epithelium from the connective tissue, producing a positive Nikolsky sign that supports a vesiculobullous process, although its absence does not exclude it [3]. Symptoms range from mild discomfort to severe burning pain, which is exacerbated by acidic, spicy, or abrasive foods and tooth brushing. This discomfort frequently discourages effective plaque control, compounding erythema through superimposed plaque-induced inflammation [5,9]. Importantly, DG itself does not cause attachment loss or alveolar bone destruction; therefore, periodontal breakdown seen in affected patients usually reflects secondary plaque accumulation rather than the underlying mucocutaneous disease [1,5].

Epidemiologically, DG shows a strong predilection for women, typically presenting in the fifth and sixth decades of life, coinciding with perimenopausal hormonal changes, although pediatric and adolescent cases have also been reported [1,2]. Across large retrospective series, mucous membrane pemphigoid (MMP), oral lichen planus (OLP), and pemphigus vulgaris (PV) account for the substantial majority of DG presentations, with published estimates attributing approximately 80% of cases to these three entities [1,3]. In a case series, MMP has been reported as the underlying diagnosis in 35-60% of DG presentations, OLP in 24-45%, and PV in 3-15%, although these proportions vary with referral patterns and the diagnostic era [1,3]. A comparative retrospective analysis spanning 55 years of biopsy records found that immunologically mediated oral diseases accounted for only 1.18% of all specimens, with lichen planus being far more common than PV or MMP; however, DG was documented across all three diagnoses and was most frequent in MMP [9]. Consistent with this, a two-center cohort comparing oral MMP with PV found DG in 84% of MMP cases versus only 28% of PV cases, while non-gingival oral lesions were far more common in PV (55%) than in MMP (6%) [7]. A separate series of 100 low-risk oral MMP cases similarly found DG as the presenting feature in 97% of patients, most often confined to a single oral site [10]. Collectively, these figures indicate that when DG is confined to the gingiva without other mucosal or cutaneous involvement, MMP should be ranked high on the differential diagnosis list, whereas widespread non-gingival oral disease should raise suspicion of PV.

The diagnostic course of DG is often prolonged. A cohort study of 41 patients presenting primarily with DG found an average patient delay of 10.8 months before the first professional consultation, followed by a further professional delay averaging 7.3 months, involving consultation with more than four healthcare providers before a definitive diagnosis was reached [4]. Less severe disease and female sex were both associated with longer patient-side delays, while professional delay was correlated with the number of clinicians consulted and the absence of extragingival lesions that might otherwise prompt an earlier suspicion of a systemic mucocutaneous disorder [4]. This prolonged diagnostic odyssey underscores the pivotal position of the general dental practitioner (GDP), who is frequently the first clinician to examine these lesions and, therefore, bears substantial responsibility for initiating a timely and structured diagnostic pathway [4,5].

Etiology and pathogenesis

The etiological spectrum underlying DG is broad, spanning autoimmune dermatoses, lichenoid disease, endocrine disturbance, hypersensitivity reactions, and chronic infection; however, the overwhelming majority of cases converge on three immune-mediated conditions: OLP, MMP, and PV [1,11]. OLP is a T-cell-mediated mucocutaneous disease in which cytotoxic lymphocytes target the basal keratinocytes. When confined to the gingiva, it typically presents as an erosive or atrophic variant rather than the more familiar reticular and asymptomatic form observed in the buccal mucosa [11,12]. Lichenoid reactions clinically indistinguishable from OLP can also be triggered by dental restorative materials, particularly amalgam, or by systemic medications; therefore, a history of recent drug changes or contact with suspected materials should be obtained [3].

MMP and PV are characterized by autoantibody-mediated subepithelial and intraepithelial blistering. In MMP, autoantibodies target basement membrane zone antigens including BP180, BP230, and laminin-332, producing subepithelial separation, while in PV, autoantibodies against desmogleins disrupt keratinocyte cell-to-cell adhesion, producing intraepithelial acantholysis [3,6]. This mechanistic distinction has direct clinical relevance because MMP carries a risk of scarring at extraoral mucosal sites, particularly conjunctival scarring that can progress to blindness, whereas PV, although potentially more acutely severe and mortality-associated when systemic, does not typically scar [3,6]. Beyond these three dominant entities, a minority of DG cases are related to plasma cell gingivitis, CUS, lupus erythematosus, erythema multiforme, linear IgA disease, epidermolysis bullosa acquisita, graft-versus-host disease, and drug- or contact-induced lichenoid lesions, several of which lack precise prevalence data owing to their rarity [1,3].

Emerging evidence also implicates the oral microbiome as a modulating, rather than purely secondary, factor in DG-associated disease. Dysbiosis, characterized by reduced microbial diversity and an increase in proinflammatory bacterial genera, has been consistently observed in OLP. This dysbiosis is correlated with epithelial barrier disruption and increased cytokine production. Proposed mechanisms include Toll-like receptor activation and skewing of T-cell responses, which may sustain mucosal inflammation [12]. Evidence in MMP and PV remains preliminary, but a shared theme across these conditions is that local microbial ecology and immune dysregulation may interact bidirectionally, indicating that plaque control is unlikely to be a purely cosmetic adjunct and may influence disease activity [12,13]. For GDP, recognizing this dual burden, an immune-mediated disease process compounded by a plaque-driven inflammatory overlay, reframes oral hygiene reinforcement as a therapeutic rather than an incidental intervention.

Differential diagnosis

Because the clinical appearance of DG is shared across multiple diseases, several less common but clinically important mimics merit specific consideration, including OLP, MMP, and PV. Chronic ulcerative stomatitis (CUS) is a rare immune-mediated disorder that closely resembles erosive OLP, both clinically and histologically. It most often affects middle-aged and older women, with cutaneous involvement in approximately one-fifth of the cases [14,15]. The key distinguishing features are serological and immunopathological, rather than clinical. CUS is characterized by stratified epithelium-specific antinuclear antibodies directed against the ΔNp63α isoform of p63, which are detectable on direct immunofluorescence (DIF), and by a comparatively poor response to conventional corticosteroids, with better outcomes reported for hydroxychloroquine [14,15]. This distinction is important because a patient labeled as having corticosteroid-refractory erosive OLP may have unrecognized CUS, requiring a different systemic agent [15].

Lichen planus pemphigoides (LPPs) represent a further diagnostic pitfall, combining the features of lichen planus with a subepithelial autoimmune blistering process. Oral involvement alone is rare, and diagnosis requires correlating clinical findings with histopathology showing features of both lichen planus and pemphigoid, supported by DIF, which demonstrates linear IgG and C3 deposition in the basement membrane zone [16]. A case series of four patients with oral LPP found that three had exclusive oral involvement, reinforcing that a gingival lesion resembling lichen planus should not automatically be assumed to be uncomplicated OLP if the biopsy features are atypical [16]. Beyond these entities, the dermatologic oral examination literature emphasizes that site-based pattern recognition, correlating the anatomical distribution of oral lesions with a structured differential, meaningfully narrows the diagnostic possibilities before biopsy is even undertaken, since certain conditions have characteristic zones of predilection within the oral cavity [13].

Because the clinical overlap among OLP, MMP, PV, CUS, and LPP is substantial and biopsy is not always immediately accessible, a table summarizing the principal differentiating features of the major DG-associated entities is a practical aid for GDPs navigating an unfamiliar presentation. Table 1 summarizes the epidemiological, clinical, and histopathological features that help distinguish the principal diseases underlying DG. Given this overlap, the GDP's task is not to render a definitive histologic diagnosis but to recognize the DG pattern, exclude simple plaque-induced gingivitis, and initiate a structured referral for tissue-based confirmation.

**Table 1:** Differential diagnostic features of the principal causes of desquamative gingivitis Abbreviations: DIF, direct immunofluorescence; IgG, immunoglobulin G; C3, complement component 3; DG, desquamative gingivitis; SES-ANA, stratified epithelium-specific antinuclear antibodies Source: Original table created by the authors based on data synthesized from references [1,3,7,10,11,14-19]

Condition	Typical demographic and relative frequency	Key clinical clue	Confirmatory test
Oral lichen planus (OLP)	Women, 5th–6th decade; ~24–45% of DG cases	Reticular white striae elsewhere in mouth; gingival form often erosive/atrophic	Histopathology + DIF if erosive/diffuse erythematous
Mucous membrane pemphigoid (MMP)	Women, mean age ~66 years; ~35–60% of DG cases, often gingiva-only	Desquamative gingivitis often the sole or presenting site (84–97% of MMP cases); risk of ocular scarring	DIF showing linear IgG/C3 at basement membrane zone
Pemphigus vulgaris (PV)	Similar age range; ~3–15% of DG cases	Widespread non-gingival oral erosions common (55% of cases); systemic therapy almost always required	DIF showing intercellular IgG deposition
Chronic ulcerative stomatitis (CUS)	Middle-aged to older women; rare	Resembles erosive OLP; poor response to corticosteroids	DIF for stratified epithelium-specific antinuclear antibodies (SES-ANA)
Lichen planus pemphigoides (LPP)	Middle-aged adults; rare, may be oral-only	Mixed Lichen Planus and vesiculobullous features	Histopathology plus DIF showing linear IgG/C3

Diagnostic workup for the general dental practitioner

For GDP, the diagnostic workup of suspected DG begins with a structured clinical assessment before any invasive steps are considered. A validated approach involves three sequential stages: intraoral examination of lesion morphology, location, and Nikolsky sign; a detailed history covering onset, systemic health, current infections, topical product exposure, and medication changes; and assessment of extraoral involvement of the skin, eyes, nose, or genital mucosa [1]. This staged history-taking framework was shown in a pilot study of dental undergraduates to meaningfully improve diagnostic accuracy compared with unstructured routine assessment, supporting its transferability to general practice [1].

When the clinical picture indicates an immune-mediated process, a biopsy is the pivotal next step. However, histopathology alone is frequently insufficient, and the diagnostic yield is substantially improved when paired with DIF, particularly for erosive, ulcerated, or diffusely erythematous presentations [17]. The French national guidelines for OLP explicitly recommend histological biopsy in the absence of reticular lesions and DIF biopsy for ulcerated, erosive, bullous, or diffuse erythematous gingival presentations [17]. A systematic review evaluating immunofluorescence performance found that DIF substantially outperformed indirect immunofluorescence, with sensitivity and specificity of 87.8% and 100% for PV, 92% and 98% for MMP, and 81% and 98.9% for DG-associated oral ulceration overlapping with OLP, while DIF sensitivity for OLP itself was more moderate at 64.3% with 88% specificity [19].

The biopsy site and technique significantly affected the diagnostic yield. A large meta-analysis of gingival biopsies for MMP and PV with gingival expression found DIF positivity rates of 100% for PV and 90.2% for MMP when biopsies were taken from the gingiva. The authors concluded that the biopsied site should be selected primarily for tissue quality and lesion proximity rather than a fixed anatomical rule, favoring healthy tissue adjacent to active lesions [20]. Complementing this, a retrospective comparison of biopsy techniques found that a normal buccal punch biopsy from uninvolved mucosa was statistically equivalent in sensitivity to a perilesional biopsy for both PV and multisite MMP and was more sensitive than histology or indirect immunofluorescence serology alone, offering GDPs and specialists a technically simpler alternative to perilesional sampling [18]. Because the gingival epithelium is particularly fragile and prone to shearing during conventional punch or scalpel biopsy, a dedicated stab-and-roll technique was developed specifically to preserve epithelial integrity. In a retrospective review of 27 DG cases, this method maintained intact epithelium in 51 of 52 biopsy specimens (98.1%), enabling a definitive diagnosis of MMP, PV, or OLP in most cases [21]. Collectively, this evidence supports the referral of suspected DG for biopsy incorporating both routine histopathology and DIF, ideally from perilesional or adjacent normal-appearing gingival or buccal tissue rather than frankly ulcerated areas, since severely inflamed or ulcerated tissue frequently yields non-diagnostic or false-negative immunofluorescence results [20,21].

Management and referral pathway

The management of DG operates on two complementary tracks: disease-specific pharmacotherapy directed at the underlying immune process, generally initiated and monitored by an oral medicine specialist or dermatologist, and non-pharmacological periodontal care, which falls squarely within the GDP's scope and materially affects patient comfort and disease activity, regardless of the causative diagnosis [2,5]. A systematic review of pharmacological treatments for DG identified topical clobetasol and tacrolimus as the most frequently studied and consistently effective first-line agents, both of which produced significant reductions in pain and lesion severity. Emerging adjunctive options, such as platelet-rich plasma and bioadhesive propolis- or nano-vitamin-based gels, have shown promising but preliminary results [2]. Systemic agents carry a materially higher risk profile, including candidiasis and hemolytic complications, reinforcing that escalation beyond topical therapy should occur under specialist supervision rather than general practice [2].

The evidence base for non-pharmacological, GDP-deliverable care is well-established. A systematic review of oral hygiene instruction and professional prophylaxis across nine studies involving 224 patients with OLP, MMP, plasma cell gingivitis, and PV found statistically significant reductions in pain, lesion extent, gingival bleeding, and plaque scores following structured hygiene instruction and supragingival scaling, regardless of the underlying diagnosis [22]. Reported protocols emphasized a modified Bass brushing technique with a soft brush, chlorhexidine mouth rinse, and avoidance of sodium lauryl sulfate-containing toothpaste, with benefits demonstrated as early as one week and sustained improvement through 20 weeks of follow-up in several cohorts [22]. Clinical case reports further support guided biofilm therapy using erythritol-based air-polishing powders as a gentler, better-tolerated alternative to conventional mechanical debridement in patients with MMP, PV, and OLP, reducing procedural discomfort while achieving effective biofilm removal in an already fragile and painful gingival environment [23]. Comprehensive reviews of clinical findings in DG similarly stress that untreated plaque accumulation, itself a consequence of the pain-driven avoidance of oral hygiene, can independently drive periodontitis and tooth loss in these patients, making professional plaque control an essential rather than optional component of care [8].

Practically, once DG is suspected, the GDP's role is to perform an atraumatic biopsy or arrange prompt referral for one, initiate gentle, individualized oral hygiene reinforcement using non-abrasive techniques, and establish a referral pathway to oral medicine, periodontology, or dermatology for definitive diagnosis and disease-specific pharmacotherapy [5,22]. Given the risk of ocular scarring in MMP and the potential for systemic morbidity across several of these conditions, referral should not await pharmacological failure but should be initiated promptly upon recognition of a DG pattern, in parallel with the institution of supportive periodontal care [5,6].

## Conclusions

DG is a clinical sign rather than a diagnosis, most often signaling oral lichen planus, mucous membrane pemphigoid, or pemphigus vulgaris, although several rarer immune-mediated conditions can produce identical gingival appearances. For general dental practitioners, the diagnostic priority is pattern recognition rather than definitive labeling; a structured history and examination addressing lesion morphology, distribution, and extraoral involvement can substantially narrow the differential diagnosis before tissue is obtained. Because histopathology alone frequently underdiagnoses these conditions, biopsy planning should routinely incorporate direct immunofluorescence sampling from perilesional or uninvolved tissue using an atraumatic technique that preserves the fragile gingival epithelium. Diagnostic delay remains common and clinically consequential; therefore, prompt referral to oral medicine, periodontology, or dermatology should not be deferred pending trial therapy. Meanwhile, GDP can meaningfully improve patient comfort and disease activity through gentle, individualized oral hygiene reinforcement and professional plaque control, interventions supported by consistent evidence regardless of the underlying diagnosis. A working knowledge of this practical pathway equips general practitioners to shorten the diagnostic journey and serve as active partners rather than passive referrers in the long-term care of these patients.
